# A multi-strain probiotic formulation preserves intestinal epithelial and vascular barriers during enteropathogenic infection

**DOI:** 10.3389/fmicb.2025.1631322

**Published:** 2025-07-25

**Authors:** Anna Maria Naso, Michela Lizier, Carmen Correale, Alessandra Silvestri, Giuseppe Penna, Paola Brescia, Maria Rescigno

**Affiliations:** ^1^IRCCS Humanitas Research Hospital, Rozzano, Italy; ^2^Department of Biomedical Sciences, Humanitas University, Pieve Emanuele, Italy

**Keywords:** gut vascular barrier, microbiota, leaky-gut, probiotics, epithelial barrier integrity

## Abstract

**Background:**

The integrity of the intestinal barrier, composed progressively of a mucus, epithelial and vascular layer is critical for maintaining gut homeostasis and preventing systemic translocation of pathogens. Disruptions in any of these protective layers can lead to various health issues, highlighting the need for strategies to preserve barrier function. This study investigated the effects of a multi-strain probiotic formulation (MPF), on intestinal barrier integrity in a murine model infected with *Salmonella typhimurium*.

**Methods:**

C57BL/6 mice were pre-treated with MPF for 10 days before oral infection with Salmonella. Intestinal barrier integrity was assessed through histological analysis, immunofluorescence for key barrier proteins, quantification of bacterial translocation and morphological changes in the ileum and colon.

**Results:**

Mice pre-treated with the probiotic formulation exhibited a preserved mucus layer, maintained intestinal epithelial barrier (IEB) integrity, evidenced by sustained expression of mucins and the tight junction protein Zonula occludens-1 (ZO-1), and reduced *Salmonella* translocation in the colon. Furthermore, the MPF maintained the gut vascular barrier (GVB) integrity by preventing the upregulation of plasmalemma vesicle-associated protein-1 (PV1), typically induced by Salmonella infection. The treatment also mitigated morphological damage, including villus and crypt shortening, caused by the pathogen.

**Conclusion:**

These findings suggest that this new formulation of multi-strain probiotics protects against *Salmonella*-induced damage to both the IEB and GVB, supporting its potential as a therapeutic intervention for managing conditions associated with intestinal barrier dysfunction. Further research is warranted to elucidate the specific mechanisms of action and validate these results in human populations.

## Introduction

The human body is inhabited by a diverse community of microorganisms, which play a vital role in our overall well-being. One of the body’s first lines of defense against harmful pathogens is the intestinal epithelial barrier (IEB), which regulates interactions between host cells and the gut microbiota.

Maintaining the integrity of the IEB is essential for homeostasis, assuring the functionality of the differentially specialized intestinal epithelial cells, linked by tight junctions (TJs) to form a continuous monolayer which is composed of various proteins, including claudin family members and junctional complex proteins such as zonula occludens (ZO-1) (Odenwald and Turner, 2017). A crucial component of this barrier is the overlying mucus layer, secreted by goblet cells, which traps pathogens and prevents their direct contact with epithelial cells. This mucus also provides a habitat for commensal bacteria, reinforcing host-microbe symbiosis (Paone and Cani, 2020).

Recent studies have identified an additional protective layer beyond the intestinal epithelium, known as the Gut Vascular Barrier (GVB). Structurally and functionally analogous to the blood-brain barrier (BBB), the GVB consists of endothelial cells supported by pericytes and enteric glial cells, forming a specialized gut-vascular unit. While more permissive than the BBB, allowing the diffusion of molecules up to ∼4 kDa, the GVB however, acts as a selective filter, enabling antigen sampling and immune tolerance, while blocking bacterial translocation in homeostatic conditions (Spadoni et al., 2015, 2017). A key regulator of GVB integrity is the canonical Wnt/β-catenin signaling pathway. Disruption of this pathway has been linked to GVB breakdown in various diseases (Spadoni et al., 2015). For instance, infections by the enteropathogenic *Salmonella enterica* serovar Typhimurium have been shown to compromise the integrity of the GVB, facilitating the dissemination of bacteria to the liver and spleen in orally infected mice. This disruption is associated with reduced β-catenin target gene expression and upregulation of plasmalemma vesicle protein-1 (PV1), which is a structural component of stomatal and fenestral diaphragms in blood vessels and regulates the permeability of endothelial cells. Conversely, endothelial expression of a constitutively active β-catenin restores GVB sealing, reduces PV1 levels, and prevents systemic spread of bacteria (Spadoni et al., 2015).

Beyond enteric infections, GVB dysfunction has been implicated in a range of chronic pathological conditions, particularly those associated with inflammation and microbial dysbiosis. In non-alcoholic fatty liver disease (NAFLD), gut dysbiosis has been shown to trigger early GVB disruption, preceding hepatic inflammation and steatosis. Restoration of GVB integrity, via endothelial β-catenin activation or pharmacological treatment with obeticholic acid (OCA), ameliorates liver pathology (Mouries et al., 2019). Similarly, increased PV1 and GVB leakiness have been documented in alcoholic liver disease (Grander et al., 2020) and ankylosing spondylitis (Ciccia et al., 2017), where systemic dissemination of gut-derived lipopolysaccharide (LPS) promotes chronic inflammation. In colorectal cancer (CRC), GVB dysfunction is associated with metastasis formation. High PV1 in colonic endothelium correlates with liver bacterial load and pre-metastatic niche formation (Bertocchi et al., 2021). Dysbiosis-driven GVB impairment can also be transferred via fecal microbiota transplantation, further supporting its causal role (Mouries et al., 2019).

These findings highlight the GVB as a crucial checkpoint in maintaining compartmentalization of gut contents. Its disruption links intestinal dysfunction to systemic diseases, from liver disorders to neuroinflammation (Brescia and Rescigno, 2021).

In response to these challenges, probiotics have emerged as a potential solution for gut-related issues, enhancing gut barrier function and restoring homeostasis. Through modulation of tight junctions, regulation of mucin production, and suppression of pathogenic bacteria, probiotics improve IEB integrity and reduce inflammation (Zhou et al., 2022). However, their impact on the GVB has not yet been elucidated.

One promising probiotic formulation (named multi-strain probiotic formulation, MPF), combines different strains of beneficial bacteria (*Lactobacillus rhamnosus* - Strain LR32, *Bifidobacterium lactis* - Strain BL04, *Bifidobacterium longum* - Strain BB536), with key nutrients (L-Teanine, L-Cistine, vitamin B2 and B3) to support healthy intestinal function.

This study aims to assess the effects of this multi-strain probiotic formulation on intestinal barrier preservation, both IEB and GVB, in a murine model of *Salmonella* infection. This research could pave the way for a novel probiotic formulation to restore host-microbe equilibrium and mitigate the impacts of a leaky gut.

## Materials and methods

### Test material

Serobioma is a food supplement formulation provided by Bromatech s.r.l., Milan, Italy. It belongs to the category of supplements for intestinal functionality, containing L-Teanine, L-Cistine, vitamin B2 and B3 lyophilized probiotic species (*Lactobacillus rhamnosus* Strain LR32, *Bifidobacterium longum* Strain BB536, and *Bifidobacterium lactis* Strain BL04). Fresh bacterial cultures were prepared daily and dissolved in water before administration in mice.

### Salmonella infection model and treatment

*Salmonella enterica* serovar Typhimurium strain SL3261AT, grown at 37°C in Luria-Bertani (LB) broth, is an aroA-metabolically defective strain on SL1344 background characterized by an attenuated ability to replicate *in vivo* due to aromatic amino acid auxotrophy (Avogadri et al., 2005). It was genetically modified by transformation with a plasmidic DNA carrying both mCherry fluorescent reporter gene and Chloramphenicol Acetyltransferase (CAT) gene, which provides resistance to chloramphenicol.

C57BL/6J mice were purchased from Charles River laboratories and maintained on a 12-h light-dark cycle with controlled temperature and under specific pathogen-free conditions. All experiments were performed in accordance with the guidelines established in the Principles of Laboratory Animal Care, approved by the Italian Ministry of Health (47/2025-PR) and consistent with national (D.L. N. 26, G.U. March 4, 2014) and international law and policies (EEC Council Directive 2010/63/EU).

Mice were treated daily with Serobioma probiotics for a period of 10 days (10^8^ CFUs/strain) via oral gavage. The selected dose was based on previous studies demonstrating barrier-protective and immunomodulatory effects of probiotics in murine models, with similar or lower doses (10^6^–10^9^ CFUs) shown to be effective in preserving intestinal integrity and modulating host responses (Chandrasekaran et al., 2024; DiMattia et al., 2024). Following pretreatment, mice were infected with 10^9^–10^11^ CFU of *Salmonella* via oral gavage and after 6–16h they were euthanized.

Colons were aseptically removed and incubated 30 min at 37°C with gentamycin to kill external bacteria and subsequently digested with 1 mg/ml Collagenase D (Roche) for 30 min at 37°C. Cells isolated from the colon were lysed with 0.5% sodium-deoxycholate and plated on LB agar with chloramphenicol to evaluate *Salmonella* dissemination after overnight culture.

### Intestinal tissue processing (H&E and Alcian blue/PAS staining)

Intestinal tissues (ileum and colon) were dissected and fixed with 10% formaldehyde, embedded in paraffin, stained with hematoxylin and eosin (H&E) and then subjected to blinded histological assessment. Alcian Blue/PAS (Periodic Acid Schiff) staining technique was used to detect acid (Alcian Blue) and neutral (PAS) mucins on colon FFPE samples (4 μm) according to Alcian Blue/PAS Stain manufacture’s instruction (Thermo Fisher Scientific #87023; Waltham, Massachusetts). Briefly, FFPE samples were deparaffinized with xylene and rehydrated with alcohol. Sections were stained in Alcian Blue pH 2.5 for 30 min at room temperature (RT), followed by a wash in tap water for 5 min and oxidation in Periodic Acid Solution for 5 min at RT. Sections were rinsed in 3 changes of distilled water and stained in Schiff Reagent for 15 min at RT. Nuclei were counterstained with Mayer’s hematoxylin (Histo-Line Laboratories; Milan, Italy). Tissues were mounted with Eukitt, and acquired with an Olympus BX51widefield microscope, 20× objective. The length of villi in the ileum and crypt depth in the colon were measured microscopically using Fiji/ImageJ software for image analysis.

### Immunofluorescence

Mouse intestinal tissues were fixed overnight in PLP buffer (1% paraformaldehyde, L-lysine 0.2 M pH 7.4, and 25 mg NaIO4). After fixation, the tissues were transferred to sucrose 20% for at least 4 h and then embedded in optimum cutting temperature compound (OCT) and stored at −80°C. For MUCIN-2 staining, 10-μm cryosections were heated in 10 mM citric acid (pH 6.0) in 800W microwave (4 × 5 min) for antigen retrieval, blocked with 0.1 M Tris-HCl pH 7.4, 2% fetal bovine serum (FBS), 0.3% Triton X-100 and stained with recombinant anti-MUC2 antibody [EPR23479-47] (antibody dilution 1:500; Abcam #272692; Cambridge, UK) overnight. Donkey anti-rabbit IgG Alexa Fluor 488 (antibody dilution 1:1000, Thermo Fisher Scientific #A31572; Waltham, Massachusetts) was then applied for 2 h at RT. For IEB and GVB staining, 10-μm cryosections were rehydrated and blocked with 0.1 M Tris-HCl pH 7.4, 2% FBS, 0.3% Triton X-100. Sections were stained with the following antibodies: monoclonal antibody anti-ZO1 [ZO1-1A12], Alexa Fluor-488 (antibody dilution 1:100; Invitrogen #339188; Waltham, Massachusetts); monoclonal antibody anti-CD34 [RAM34], Alexa Fluor 647 (antibody dilution 1:50; Bioscience #560230; Franklin Lakes, New Jersey) and monoclonal antibody anti-PV1 [MECA32] (antibody dilution 1:100; BD Pharmingen #553849; Franklin Lakes, New Jersey) with appropriate fluorophore-conjugated secondary antibody, for 2h at RT. Nuclei were counterstained with 4′, 6-diamidino-2-phenylindole (DAPI). Sections were then mounted with Vectashield Antifade (Vector Laboratories; Burlingame, California). Acquisition by confocal microscopy was performed on a Leica SP8 (oil objective) at 40× magnification and Fiji (ImageJ) software package used for image analysis and fluorescence quantification.

### Statistical analysis

Results are expressed as mean ± SE. Statistical analysis was carried out with the computer-assisted Prism GraphPad program (Prism version 8.1; GraphPad Software, San Diego, CA, USA). Unpaired *T*-test was used for paired comparisons. *P*-values < 0.05 were considered significant.

## Results

To explore the impact of MPF on intestinal epithelial and endothelial barrier integrity, mice were pretreated for 10 days with a combination of *Lactobacillus rhamnosus* - Strain LR32, *Bifidobacterium lactis* - Strain BL04, *Bifidobacterium longum* - Strain BB536, followed by oral infection with *Salmonella typhimurium* SL3261AT, a mutant strain retaining invasiveness but with attenuated intracellular survival (Martinoli et al., 2007).

**FIGURE 1:**
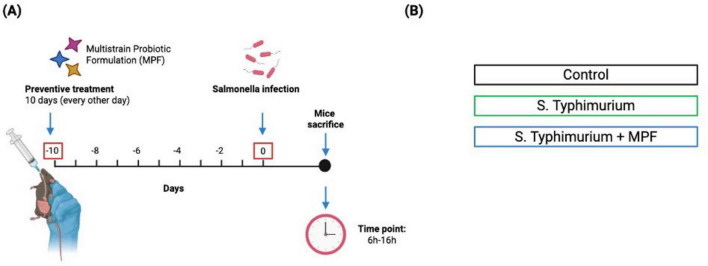
Schematic representation of experimental design. **(A)** Timeline of *Salmonella typhimurium* infection with multi-strain probiotic formulation (MPF) treatment, showing pre-exposure (10 days) and post-infection period (6–16 h). **(B)** The experimental groups included: Control Group (not exposed to *S. Typhimurium* or multi-strain probiotic formulation-MPF), Infected Group (exposed to *S. Typhimurium* alone), Treatment Group (exposed to both *S. Typhimurium* and MPF). Created with Biorender.

Mice were randomly divided in 3 experimental groups: not infected controls, *Salmonella-*infected without MPF, and *Salmonella*-infected with MPF pretreatment (Figure 1). Animals were euthanized 6–16h post-infection, to assess both acute and intermediate barrier integrity changes. Previous findings showed that intestinal damage and bacterial translocation significantly decrease beyond 16–24 h of infection (Spadoni et al., 2015). As such, later time points are less informative in this model and were not included to maintain focus on the initial protective window of the probiotics.

Throughout the MPF pre-treatment and the infection period, no significant differences in body weight or observable clinical symptoms (such as reduced movement, hunched posture or piloerection) were observed among the experimental groups. These findings confirm that neither MPF administration nor infection with the attenuated *Salmonella* strain caused overt systemic illness (data not shown).

To evaluate barrier integrity, intestinal tissue sections were stained for mucins (major structural and functional components of the mucus), the epithelial tight junction protein ZO-1, and the endothelial permeability marker PV1.

**FIGURE 2:**
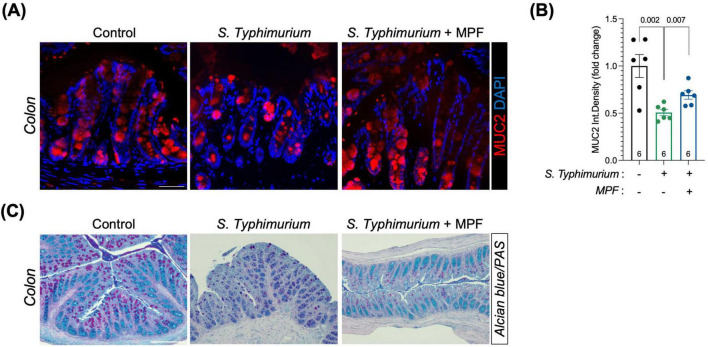
Multi-strain probiotic formulation (MPF) supports intestinal mucus layer retention against *Salmonella* challenge. **(A)** Colon tissue sections (harvested after 6 h of infection) stained for MUC2 (red) and DAPI (blue). Representative images of a single mouse of six for each experimental condition. Scale bar 50 μm. **(B)** Quantification of MUC2 fluorescent signal as integrated density (six mice per group), performed using Fiji software. **(C)** Colon tissue sections stained with Alcian Blue/Periodic Acid-Schiff (PAS). Scale bar 100 μm. Line at mean with SEM; statistical analysis was evaluated using unpaired *t*-test.

In the colon, *Salmonella*-infected mice showed an impairment in the mucus layer, indicated by a pronounced loss in mucin expression, as measured by MUC2 (Figures 2A, B) and histological Alcian Blue/PAS staining (Figure 2C). In contrast, MPF-treated mice maintained robust MUC2 signal intensity and mucus layer structure comparable to not infected controls. Quantification confirmed a significant preservation of mucus in the MPF group, indicating that probiotic pre-treatment contributes to enhanced defense against bacterial access to the underlying epithelial monolayer.

**FIGURE 3:**
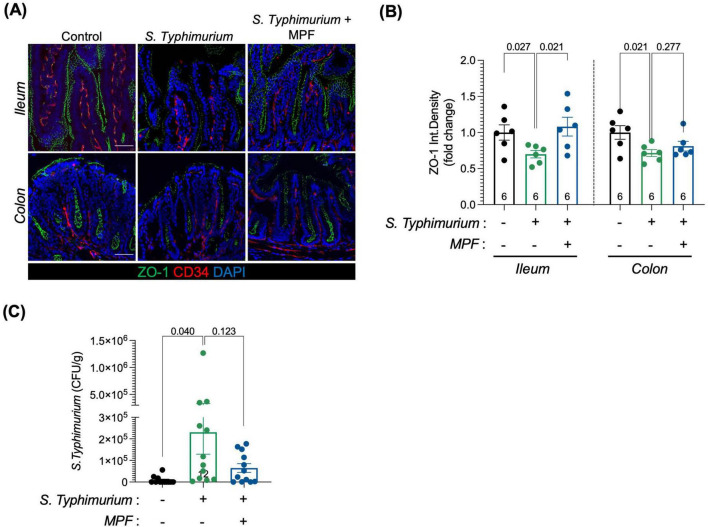
Multi-strain probiotic formulation (MPF) helps preserving the epithelial layer of the intestinal barrier upon *Salmonella* infection. **(A)** Ileum and colon tissue sections (harvested after 6 h of infection) stained for ZO-1 (green); CD34, a marker of vessels (red) and DAPI (blue). Representative images of a single mouse of six for each experimental condition. Scale bar 50 μm. **(B)** Quantification of ZO-1 fluorescent signal in CD34-neg area of ileum (left) and colon (right) expressed as integrated density (six mice per group), performed using Fiji software. **(C)** *S. Typhimurium* dissemination in the colon lamina propria after 16 h of infection (twelve mice per group), indicated as number of Colony Forming Unit per gram of tissue. Line at mean with SEM; statistical analysis was evaluated using unpaired *t*-test.

At 6 h post-infection, *Salmonella*-induced epithelial barrier disruption was confirmed by reduced ZO-1 levels in the ileum and colon. Interestingly, treatment with MPF maintained ZO-1 expression at levels comparable to controls, especially in the ileum (Figures 3A, B).

Assessment of *Salmonella* translocation across the intestinal barrier revealed the presence of bacteria in the colonic lamina propria in *Salmonella*-infected mice, indicating compromised integrity. This translocation was significantly reduced in MPF-treated animals (Figure 3C), supporting the role of this formulation in limiting epithelial breach and containing the infection within the intestinal lumen without deeper tissue invasion.

**FIGURE 4:**
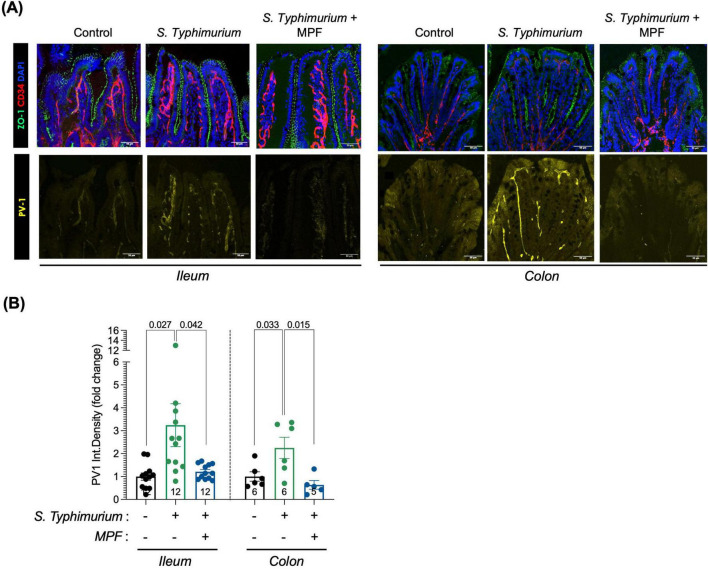
Multi-strain Probiotic Formulation (MPF) maintains GVB integrity upon *Salmonella* infection. **(A)** Ileum and colon tissue sections (harvested after 16 h of infection) stained for ZO-1 (green); PV1 (yellow); CD34 (red) and DAPI (blue). Representative images of a single mouse of six (colon) or twelve (ileum) for each experimental condition. Scale bar 50 μm. **(B)** Quantification of PV1 fluorescent signal in CD34-pos area, expressed as integrated density (fold change on control group; number of mice per group indicated inside the bar), performed using Fiji software. Line at mean with SEM; statistical analysis was evaluated using unpaired *t*-test.

The disruption of the intestinal epithelial layer, which is in close contact with the intestinal lumen, is one of the earliest events triggered by enteropathogenic infections. However, the intestinal epithelium is known for its rapid regenerative ability, which allows for partial repair shortly after the initial damage. This is likely reflected in the increased ZO-1 expression observed in the *Salmonella*-infected group at 16 h post-infection (Figure 4A). The early loss of ZO-1 at 6 h suggests initial barrier disruption, while the later rise likely represents a compensatory response as the tissue begins to heal. In contrast, ZO-1 levels in the MPF-treated group remain stable and well-organized at both time points, suggesting that the MPF helped maintain normal barrier structure.

Conversely, we observed a statistically significant increase in PV1 marker levels in endothelial cells (CD34-positive cells, depicted in red in Figure 4A), indicating a marked GVB disruption, after 16 h of *Salmonella* infection. Interestingly, supplementation with MPF maintains PV1 in the GVB at levels comparable to the not-infected control group, indicating a preserved integrity of the endothelial barrier both in the ileum and colon (Figures 4A, B).

These results suggest that this new multi-strain probiotics help protect against GVB damage caused by *Salmonella* infection. The ability of this food supplement to prevent endothelial barrier disruption is particularly significant, as gut endothelial integrity is crucial for regulating the systemic spreading of inflammation and modulating immune responses during infection.

The ileum and colon of mice were histologically examined to evaluate the impact of MPF on intestinal morphology changes induced by *Salmonella* infection. Hematoxylin and Eosin (H&E) staining allowed for detailed visualization of key intestinal structures, particularly villi and crypts, which serve as important indicators of gut health. Measurements of villus length and crypt depth were used to evaluate the extent of damage and the efficacy of MPF in preserving tissue integrity.

**FIGURE 5:**
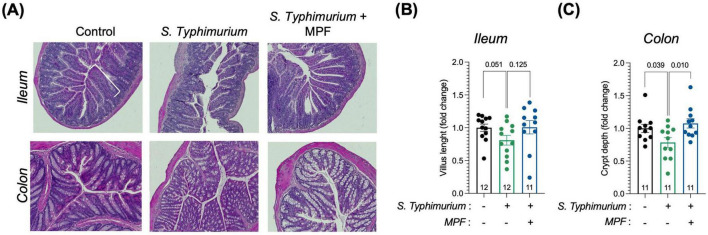
Intestinal tissue morphology is preserved by a Multi-strain Probiotic Formulation (MPF) after Salmonella infection. **(A)** Panel represents histology analysis performed on ileum and colon FFPE sections: H&E staining (upper panel). Scale bar 20 μm. **(B,C)** Quantification of villus length **(B)** and crypt depth **(C)** in the ileum and colon, respectively, performed using Fiji software (fold change on control group). Line at mean with SEM; statistical analysis was evaluated using unpaired *t*-test.

In *Salmonella*-infected mice, a mild reduction of villus length in the ileum and crypt depth in the colon was observed. Interestingly, mice treated with the MPF displayed an average villus length and crypt depth comparable to the not infected group, although a higher variability was observed in the ileum, as illustrated in the representative images (Figure 5A) and quantification in the graph (Figures 5B, C). This suggests that the probiotics can mitigate the intestinal morphological alterations caused by *Salmonella*, indicative of acute intestinal damage.

These findings indicate that the new multi-strain probiotic formulation tested may confer protective properties to the intestinal barrier. By maintaining the integrity of the intestinal tissue, these probiotics could play a crucial role in promoting gut health during and after an infection, preventing substantial damage to the intestinal lining and reducing the risk of long-term complications if not managed effectively.

## Discussion

The findings of this study provide evidence for the protective role of a new multi-strain probiotic formulation in maintaining intestinal barrier integrity under conditions of pathogenic challenge.

The results demonstrate that this probiotic supplementation effectively counteracts the morphological disruptions caused by *Salmonella* infection, including alterations in villus length and crypt depth. Furthermore, it preserved the expression of mucins and ZO-1, a key tight junction protein critical for maintaining IEB integrity. These findings suggest that this new probiotic formulation not only prevents the initial damage induced by bacterial infection but also promotes the re-establishment of a sealed IEB, thereby reducing intestinal permeability and minimizing translocation of pathogens across it.

In addition to its effects on the IEB, this study highlights the potential of this new MPF to modulate GVB function. Prolonged exposure to *Salmonella* significantly affected PV1, a marker of vascular barrier integrity, leading to GVB disruption. Under these conditions, the probiotics maintained PV1 levels closer to those observed in non-infected controls, indicating their ability to prevent GVB dysregulation. This protective effect underscores the close link between intestinal barriers and the importance of addressing both epithelial and vascular components in managing gut barrier dysfunction.

The study also sheds light on the dynamic nature of intestinal barrier repair mechanisms. The observed differences in ZO-1 expression kinetics suggest that compensatory responses may temporarily maintain barrier function despite inflammatory insults.

However, prolonged or severe challenges can exhaust these mechanisms, resulting in persistent barrier damage. The ability of this new formulation of probiotics to stabilize both IEB and GVB under such conditions highlights its therapeutic potential in preventing the exhaustion of compensatory capacity.

*Lactobacilli* and *Bifidobacteria* are essential members of the human gut microbiota, playing crucial roles in maintaining overall health through their immune-modulating properties and their contribution to the integrity of tight junctions within the intestinal epithelium (Sarita et al., 2024). *Bifidobacterium longum* BB536 is particularly noted for its ability to balance gut microbiota, restore mucus growth (Schroeder et al., 2018) and enhance the immune response, with clinical trials showing its effectiveness in alleviating symptoms of ulcerative colitis (Akatsu et al., 2013; Tamaki et al., 2016; di Vito et al., 2022). *Lactobacillus rhamnosus* LR32 also demonstrates immunomodulatory properties and supports gut health by restoring microbial balance and increasing tight junction protein expression in animal models (Huang et al., 2023). Similarly, *Bifidobacterium lactis* BL04 is effective in improving gastrointestinal symptoms and has been shown to reduce the risk of upper respiratory infections in healthy individuals (Bartosch et al., 2005). Additionally, *Bifidobacteria* exhibit anti-inflammatory properties by lowering inflammatory cytokine levels and inhibiting harmful signaling pathways in conditions like endotoxin-induced injury and necrotizing enterocolitis (Riedel et al., 2006; Underwood et al., 2014; Gavzy et al., 2023). They produce short-chain fatty acids (SCFAs) through fermentation, which improve intestinal barrier function, reduce inflammation, and aid in healing the intestinal lining (Fukuda et al., 2011; Devika and Raman, 2019; Yoon et al., 2023). By increasing the concentration of SCFAs in the gut, *Bifidobacteria* can help restore microbial balance and mitigate the adverse effects of enteropathogens, such as *Salmonella* infections, thereby supporting overall immune function and intestinal health.

While previous studies have established that probiotics enhance tight junction integrity, modulate immune responses, and inhibit pathogen adhesion, most focus solely on epithelial protection. This study uniquely demonstrates this specific multi-strain probiotic formulation preserves both IEB and GVB integrity during *Salmonella* infection. By maintaining mucins and tight junction proteins (e.g., MUC2 and ZO-1, respectively), reducing bacterial translocation, and preventing vascular disruption (e.g., via PV1), our findings uncover a critical, yet underexplored, role for probiotics in sustaining vascular barrier function, a key advance given the interdependence of intestinal barrier layers.

The implications of these findings extend beyond acute infections. The disruption of intestinal barriers is a driving event of numerous chronic conditions, including inflammatory bowel diseases (IBD), metabolic syndrome, and neurodegenerative disorders. By preserving gut structure and function, this multi-strain probiotic formulation may offer a promising strategy for managing these pathologies. Moreover, its ability to limit bacterial translocation and potentially control systemic inflammation renders it a valuable candidate for preventing complications associated with leaky gut syndrome. For instance, one possible application of multi-strain probiotic administration is in irritable bowel syndrome where defects in IEB and GVB have also been demonstrated, with IEB damage associated with bloating, abdominal pain and increased barrier permeability, while GVB disruption is linked to anxiety and depression (Barbaro et al., 2024; Santos and Rescigno, 2024). Consistently, a recent study has shown the potential of the same MPF in counteracting the increased permeability observed in IBS patients by enhancing the expression of various TJ proteins, including ZO-1 (Barbaro et al., 2025).

Despite these promising results, several limitations and opportunities for future research should be acknowledged. This study was performed in a murine model, which, while informative for dissecting intestinal barrier mechanisms, may not fully recapitulate the complexity of human gut physiology in terms of immune responses, microbiota composition, and barrier dynamics. To strengthen translational relevance, future work should incorporate *ex vivo* human systems–such as intestinal organoids or tissue biopsies–and clinical cohorts to validate these effects in a human context. Further validation in human-relevant systems will be essential to assess the efficacy, safety, and strain-specific effects of this MPF in human settings.

Our findings demonstrate a clear structural preservation of both the intestinal epithelial and vascular barriers, however the molecular pathways underlying these effects remain not completely understood. Moreover, the gut barrier is a multi-layered system, and potential effects on the gut lymphatic barrier and mucosal immune responses were not investigated. Future studies should explore these additional compartments to fully elucidate the mechanisms underlying probiotic-mediated protection. A multi-omics approach integrating microbial profiling, host transcriptomics, cytokine analysis, and metabolomics (e.g., short-chain fatty acid quantification) will help to dissect how this probiotic formulation influences gut ecology, immune signaling, and epithelial-vascular crosstalk. These strategies will help to define not only strain-specific effects but also common downstream pathways that mediate barrier protection.

In parallel, refining the functional assessment of barrier integrity will also be relevant. PV1 was used here as a vascular permeability marker, since our previous work demonstrated its strong correlation with enhanced vascular leakage in *Salmonella* infection and other models of intestinal injury, as confirmed by functional permeability assays (e.g., FITC-dextran leakage) (Spadoni et al., 2015; Mouries et al., 2019). These studies also showed that in a genetic mouse model with endothelial-specific β-catenin activation (VecPac), PV1 was reduced and the GVB remained sealed, preventing bacterial dissemination to distal organs such as the liver. This strongly supports the use of PV1 as a surrogate indicator of vascular dysfunction. Nevertheless, future studies using functional permeability assays under MPF treatment conditions would further validate its protective effects.

Additionally, this study did not include a probiotic-only treatment group to assess the baseline impact of the formulation on an uninfected gut. However, prior work has shown that this probiotic formulation does not alter Caco-2 monolayer permeability or TJ protein expression *in vitro* (di Vito et al., 2022; Barbaro et al., 2025), and that it lacks a pro-inflammatory effect on human PBMCs (Sichetti et al., 2018). Moreover, our recent findings demonstrated that *Lactobacillus paracasei*-derived postbiotics protect intestinal epithelial cells against *Salmonella*-induced injury by modulating inflammatory and barrier-related pathways, supporting the concept that both live bacteria and their metabolic products contribute to gut barrier protection (Algieri et al., 2023).

These aspects should be explored more thoroughly in future investigations and may uncover conserved microbial-host interaction signatures and identify biomarkers of probiotic responsiveness. Furthermore, the therapeutic potential of the MPF should be tested across diverse clinical conditions associated with gut barrier dysfunction, such as inflammatory bowel diseases, metabolic disorders, and neuroinflammation.

In conclusion, this study highlights the multifaceted benefits of multi-strain probiotics in protecting gut barrier integrity during pathogenic challenges. By stabilizing both IEB and GVB functions, they mitigate immediate damage and may support long-term barrier health. These findings contribute to a growing body of evidence supporting probiotics as a viable intervention for managing diseases associated with impaired intestinal barriers. Further research into strain-specific mechanisms and clinical applications will be essential to fully address their therapeutic potential.

## Data Availability

The original contributions presented in this study are included in this article/supplementary material, further inquiries can be directed to the corresponding authors.
